# Observation of the Expression of Vascular Endothelial Growth Factor and the Potential Effect of Promoting Hair Growth Treated with Chinese Herbal BeauTop

**DOI:** 10.1155/2021/6667011

**Published:** 2021-02-17

**Authors:** Chien-Ying Lee, Chun-Hung Su, Chien-Ying Chiang, Chun-Nan Wu, Yu-Hsiang Kuan

**Affiliations:** ^1^Department of Pharmacology, School of Medicine, Chung Shan Medical University, Taichung, Taiwan; ^2^Department of Pharmacy, Chung Shan Medical University Hospital, Taichung, Taiwan; ^3^Brion Research Institute, New Taipei, Taiwan

## Abstract

Despite minoxidil and finasteride already being approved by the Food and Drug Administration (FDA) for the treatment of hair loss, it is important to identify new and innovative treatments for hair loss, such as looking for a solution in Chinese herbal medicine. One such treatment to consider is BeauTop (BT), whose primary ingredients include *Panax japonicus* (T.Nees), C.A. Mey. (Araliaceae), *Astragalus membranaceus* (Fisch) Bunge (Fabaceae), *Angelica sinensis* (Oliv.) Diels (Apiaceae), *Ligustrum lucidum* W.T. Aiton (Oleaceae), *Rehmannia glutinosa* (Gaertn.) DC. (Plantaginaceae), and *Eclipta prostrata* (L.) *L*. (Compositae). The aim of this study was to evaluate whether BT can promote hair growth in C57BL/6 mice and to investigate hair coverage, the expression of vascular endothelial growth factor (VEFG), and the numbers of hair follicles in growth phase after oral administration. A total of 12 C57BL/6 mice were divided into two groups: control group and treatment group BT. BT was administered orally as an extract at a volume of 0.6 g/kg. The control group was treated with distilled water. Each group was treated once a day for 12 consecutive days. To observe the expression of VEGF distribution, the number of hair follicles and the hair coverage were examined on days 4, 8, and 12. By comparing the treatment group and control group, we found that VEGF in the BT group on day 8 presented with a higher area percentage than the control group (*p* value = 0.003). Hair follicle counting results showed that the BT group was significantly higher than the control group on day 8 (*p* value = 0.031). Furthermore, hair coverage was shown to be significantly increased in the treatment group BT on day 8 (*p* value = 0.013). Taken together, these results suggest that Chinese medicine (BT) possesses the potential effect of promoting hair growth through VEGF expression. VEGF is considered the most important mediator for the process of angiogenesis involved in hair growth development.

## 1. Introduction

Hair is thought to be an important component of an individual's feeling of attractiveness. The significant psychological effect of hair loss has a measurably negative change in self-esteem [1]. Androgenetic alopecia (AGA) is a common form of hair loss in both men and women. Approximately 50% of the male population are affected to some extent. AGA is a physiologic androgen-stimulated condition that occurs in genetically predisposed areas of the scalp [2]. The conversion of circulating testosterone into dihydrotestosterone (DHT) is mainly by the action of 5*α*-reductase type 2 [3]. DHT plays a major role in the pathogenesis of AGA, while elevated DHT level can be observed in men with AGA [4].

There are three phases of hair growth in the cycle, including the anagen, catagen, and telogen phases [5]. The hair growth cycle is regulated by several growth factors, including vascular endothelial growth factor (VEFG) [6], epithelial growth factor (EGF) [7], insulin-like growth factor (IGF)-1 [8], and fibroblast growth factor (FGF)-5 and -7 [9]. Dysregulation of these growth factors results in hair loss.

Evidence has established the fundamental role of VEGF as a key regulator of physiological and pathological angiogenesis during cutaneous development. VEGF receptor-2 is the primary receptor for VEGF and most functional effects were mediated by VEGF [10]. Importantly, transgenic mice overexpression of VEGF in keratinocytes of hair follicles strongly induced perifollicular vascularization during hair growth cycle, led to accelerated hair regrowth after depilation, and increased hair follicle size and hair shaft diameter. These findings suggested that VEGF plays an important role in the regulation of perifollicular vascularization during hair growth [11]. In addition, hair growth is induced by topical tofacitinib, the JAK3 inhibitor, via the increased concentration of VEGF and the lowered level of inflammation [12]. Based on these findings, we hypothesized that VEGF is considered the key mediator for the process of angiogenesis involved in cutaneous development and physiological and pathological processes.

In our study, the ingredients of the Chinese herbal medicine, BeauTop (BT), included *Panax japonicus* (T.Nees) C.A. Mey. (Araliaceae), *Astragalus membranaceus* (Fisch) Bunge (Fabaceae), *Angelica sinensis* (Oliv.) Diels (Apiaceae), *Ligustrum lucidum* W.T. Aiton (Oleaceae), *Rehmannia glutinosa* (Gaertn.) DC. (Plantaginaceae), and *Eclipta prostrata* (L.) *L*. (Compositae). Reports suggest that *Panax japonicus* is an immunological modulator [13]. *Astragalus membranaceus* is well known to strengthen a host's defense system [14]. *Angelica sinensis* is commonly used to promote blood circulation in the treatment of menstrual disorders [15]. Many reports have shown the pharmacological functions of *Rehmannia glutinosa* on the blood system, anti-inflammatory, and immune system [16, 17]. *Eclipta prostrata* could effectively reduce cholesterol levels in the blood and improve the antioxidant activities in rats [18].

Research in the field of reversing hair loss still remains a challenging subject. Even though topical minoxidil solution and oral finasteride have been approved by the Food and Drug Administration (FDA) as effects treatment options for androgenetic alopecia in men, it is important to improve the therapeutic approach for hair loss by looking at Chinese herbal medicine, for example. Our previous study had shown that the mechanism of BT improving hair growth is associated with the expression, in EGF and FGF-7, BT may have a potential effect to stimulate hair growth [19].

The aim of this study was to evaluate whether the Chinese herbal medicine BT can promote hair growth through the expression of VEGF in hair follicles in C57BL/6J mice. To achieve this aim, we will examine hair coverage, expression of VEFG, and the number of hair follicles in the growth phase after oral administration.

## 2. Materials and Methods

### 2.1. Animals and Skin Collection

We chose C57BL/6 mice for our study based on our review of prior related studies [11, 20]. Female C57BL/6 mice weighing 15–20 g (six weeks old) were obtained from the National Laboratory Animal Center (Taipei, Taiwan); mice were randomly grouped and cared for in a sterilized cage independent animal unit under a 12-hour light/darkness cycle in the Animal Technology Institute Taiwan (ATIT). Mice were fed mouse chow and water *ad libitum*. The environment was maintained at a temperature of around 24 ± 2°C and the humidity range was maintained from 30 to 70%. The animals were killed by cervical dislocation under an overdose of anesthesia and the skin was harvested at the level of subcutis and stored at −80°C until further use. This study was approved by the research ethics committee of the Agricultural Technology Research Institute (No. 97011) before the study begins.

### 2.2. Preparation of Chinese Herbal Medicine Formulations

The BeauTop samples were provided by the Brion Research Institute. A mixture of *Panax japonicus*, *Astragalus membranaceus*, *Angelica sinensis*, *Ligustrum lucidum*, *Rehmannia glutinosa*, and *Eclipta prostrata* at a ratio of 7 : 9 : 7 : 10 : 7 : 10 [19] was prepared. The process of making BeauTop is described in Patent US 7,828,048 B2 (reference: Patent US 7,828,048 B2, column 4, lines 59-67). All components were purchased from Sun Ten Pharmaceutical Co. Ltd. (Taipei, Taiwan).

### 2.3. Experimental Design

#### 2.3.1. Screening the Best Formulation of Chinese Herbal Medicine by Hair Follicle Counting

A total of 12 mice of C57BL/6 were divided into 2 groups, a control group and a treatment group BT. Mice were anesthetized with 2, 2, 2-tribromoethanol (Avertin®) by intraperitoneal injection at a dose of 250 mg/kg. Hot rosin and paraffin mixture depilation were used to induce hair follicles to enter from telogen into anagen and each depilatory area was fixed upon 2 cm × 2 cm. Treatment groups (BT) were orally administrated quantitative Chinese herbal medicine extract powder at 0.6 g/kg body weight. The control group received distilled water. Each group was treated once a day for 12 consecutive days.

Three mice from each group were taken for photos and skin sampling on day 4, day 8, and day 12. The samples of skin were fixed in 10% formalin solution for at least 24 hours. Each skin texture sample was sliced into three pieces and put in the same embedding box. The sliced samples were prepared for further immunohistochemical stain and counting of hair follicle number. The number of follicles per mm^2^ was then calculated using an AxioCam ICc3 microscope at × 100 magnification.

#### 2.3.2. Analysis of Growth Factor Expression of VEGF during Treatment with Chinese Herbal Medicine

A total of 12 mice of C57BL/6 were separated into 2 groups, one control group and one treatment BT group. The protocol for this is described as Step 2.1 and the same embedding box (including 3 sliced skin samples) was prepared for growth factors such as VEGF immunohistochemical stain. Fixed sections were immunostained with antimouse VEGF (Millipore, Billerica, MA), according to the manufacturer's instructions.

### 2.4. Image Analysis

Immunohistochemical stain was used in the evaluation of growth factors on each slide. All the growth factors were stained a brown color. An Olympus microscope equipped with an AxioCam ICc3 imagine system was used to randomly screen in the field and Image-Pro Plus software was utilized to calculate the percentage of color area.

### 2.5. Statistical Analysis

The summary data are presented as the mean and standard deviation unless otherwise stated. Statistical analysis for group comparison was performed via Independent t-test analysis for a comparison between the treatment and control group (SPSS version 12.0, Claritas Inc. USA.).

## 3. Results

**Figure 1:**
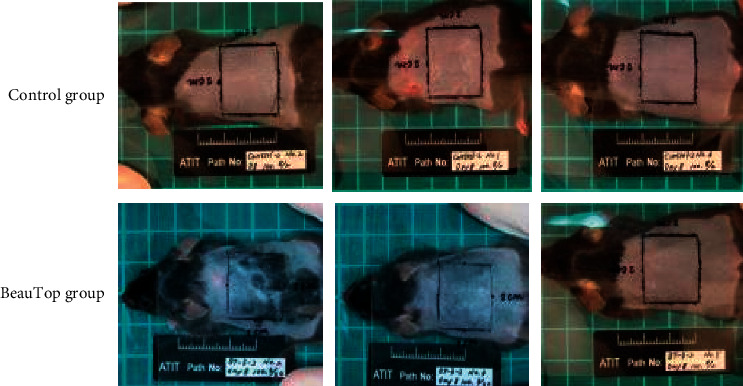
Comparison of the hair growth between control group and BeauTop group on Day 8.

We compared the treatment group and placebo group on day 8, and we observed that the accelerated hair growth in the BT group was greater than the control group (Figure 1).

**Figure 2:**
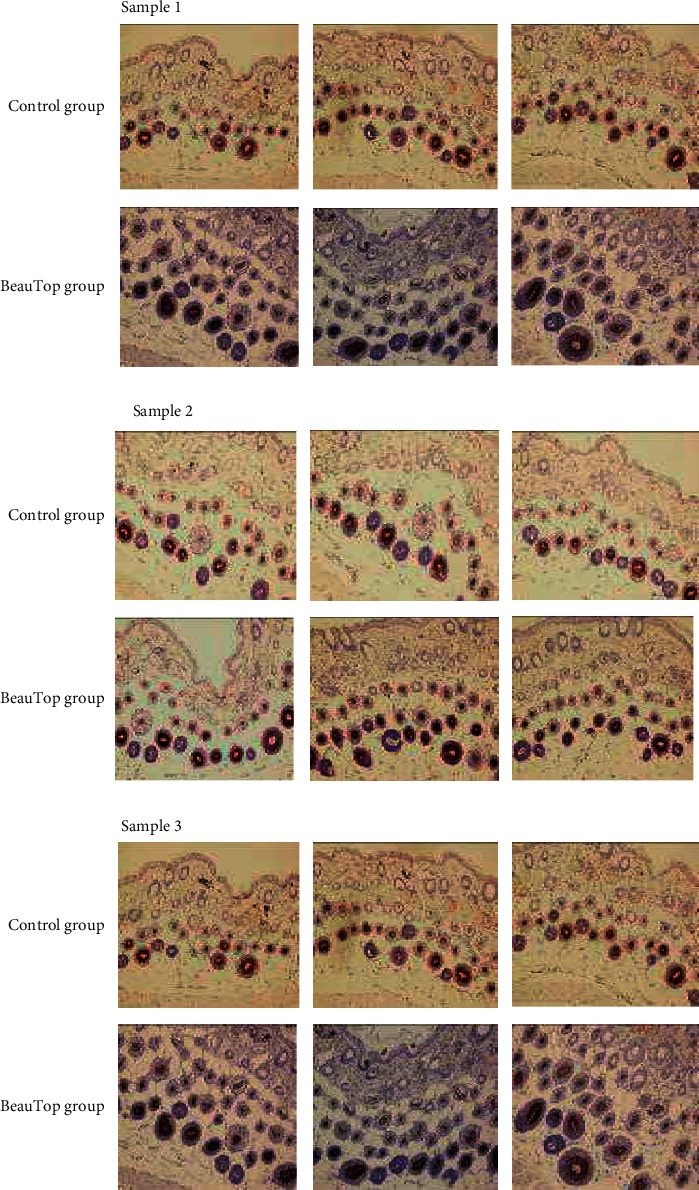
Comparison of Immunohistochemistic stain of VEGF between control group and BeauTop group on Day 8.

The immunohistochemical stain results showed that in mice treated with BeauTop, the expression in VEGF was significantly greater than the control group on day 8. In the control group, many of the hair follicles had the normal structure characteristics of the anagen phase, and both the hair bulb and the inner and outer root sheaths were clearly identified. After treatment on day 8, we observed the rapid growth of hair follicles and these enlarged primordial follicles with hypertrophy appeared to be larger in the anagen phase (Figure 2).

**Figure 3:**
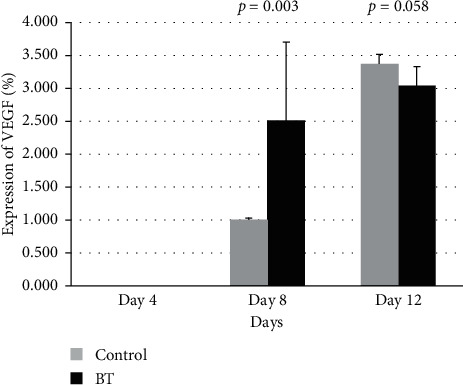
The area percentage (%) of VEGF distribution among treatment groups (BT) and control group, after being treated with the Chinese herbal medicines on Day 4, Day 8 and Day 12.

Histopathological results, comparing the treatment group and placebo group, showed that the growth factors during the study period indicated that VEGF in the BT group on day 8 presented a higher area percentage than the control group, respectively (*p* value = 0.003). The expression of VEGF did not significantly increase in BT on day 12 (*p* value = 0.058) (Figure 3).

**Figure 4:**
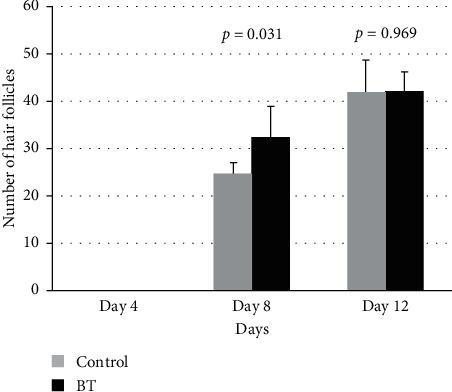
The number of hair follicle among treatment groups (BT) and control group, after treated being with the Chinese herbal medicines on Day 4, Day 8 and Day 12.

Based on prior data, we hypothesized that the BT had possessed superior hair growth effectiveness. Therefore, we took a close look at the BT formulation with regard to growth factor analysis. When examining the follicle counting results, we found that when comparing the treatment group and placebo group, the hair follicle counting in the BT group was significantly higher than the control group on day 8 (*p* value = 0.031). The difference between the control group and the BT group was not significantly on day 12 (*p* value = 0.081) (Figure 4).

**Figure 5:**
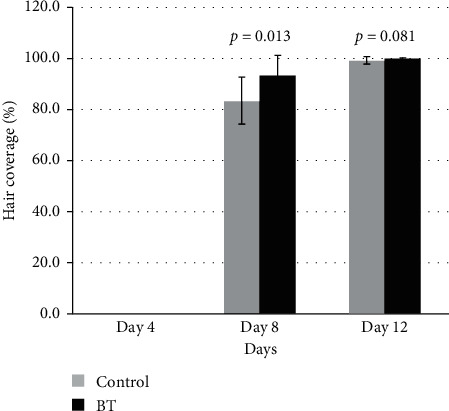
The hair coverage among treatment groups (BT) and control group, after being treated with the Chinese herbal medicines on Day 4, Day 8 and Day 12.

Hair coverage also significantly increased in the treatment group BT on day 8 (*p* value = 0.013) but did not significantly increase in the BT group on day 12 (*p* value = 0.081) (Figure 5).

## 4. Discussion

Even though minoxidil and finasteride have been approved for the treatment of hair loss, we compared Chinese herbal medicine BT treatment group and control group to explore the therapeutic effect of accelerated hair growth. Our study demonstrated that the expression in VEGF in the BT group was significantly greater than the control group on day 8, presenting a higher area percentage than the control group. We also found that hair follicle counting in the BT group was significantly higher than the control group, and hair coverage was shown to be significantly increased in the treatment group BT on day 8. Based on these findings, we suggest that BT had possessed superior hair growth effectiveness through VEGF expression, and VEGF is considered as the most important mediator for the process of angiogenesis involved in hair growth development.

Our previous study had shown that the expression of EGF and FGF-7 in the BT group was increased on day 8. However, FGF-5 in the BT group was reduced on day 12. There were no effects on the expression of IGF-1. This study indicated that BT might have a potential effect to stimulate hair growth. Taken together, the results suggest the mechanism of BT improving hair growth via the expression in EGF and FGF-7 [19]. From these data, it can be suggested that BT had a potential effect to improve hair growth via the induction of several growth factors (VEGF, EGF, and FGF), especially the expression of VEGF as the key mediator in hair growth development.

During embryonic development, VEFG is one of the most important mediators that regulate blood vessel formation on wound repair and in maintaining vascular homeostasis [21]. Deficiency in various VEGF receptors or their ligands shows serious problems in the vascular formation that causes many diseases [22]. It was demonstrated that VEGF expression in human follicles significantly decreased when compared to normal follicles [23]. From our study data, the increased expression of VEGF was significant on day 8 and presented with a higher area percentage in the treatment group as compared to the control group. It was revealed that the BT has the potential effect of stimulating hair growth via upregulation of VEGF expression. In the normal hair growth cycle, VEGF expression will stop increasing to prevent hair overgrowth, and we observed that the expression of VEGF did not significantly increase on day 12.

VEGF plays a key role in mediating the process of angiogenesis during development, and in a number of inflammatory diseases and neoplastic diseases that are associated with neovascularization [24]. In our study, we also found that the change in the number of hair follicles significantly increased the rapid growth of hair follicles and these enlarged primordial follicles with hypertrophy appeared to be larger in the BT group on day 8, revealing that BT has the potential effect of promoting VEGF expression in follicular follicles resulting in accelerated hair regrowth and increased hair follicle size in mice. As per the previous study [12], we also found similar results which show that the promotion of angiogenesis can promote hair growth through VEGF expression. We observed that the accelerated hair growth in the BT group was greater than the control group, and the hair coverage significantly increased in the treatment group BT on day 8.

A number of hair conditions are caused by hair follicles. Hair follicle affects skin conditions through the immune system and hormone system. The immune system of hair follicle is associated with the hair follicle hormone system. Certain conditions can affect hair follicles and cause hair loss. Importantly, environmental stress may influence both the immune system and hormone system of the hair follicle and lead to the induction of cell-mediated autoimmune hair disease, such as alopecia areata (AA) [25]. It is important to note that AA and alopecia universally appear to be autoimmune diseases and are not associated with androgen [26].

In our study, BT is formulated from the following six ingredients: *Panax japonicus*, *Astragalus membranaceus*, *Angelica sinensis*, *Ligustrum lucidum*, *Rehmannia glutinosa*, and *Eclipta prostrata*. One study reported the effects of saponin from *Panax japonicus* that increase the activation and synthesis of TGF-beta1 and modify the expression of TGF-beta receptors in fibroblasts [27]. A formulation containing *Panax japonicus* and *Panax notoginseng* (Burkill) F. H. Chen (Araliaceae) can promote the secretion of VEGF and the expression of VEGF receptor 2, which indicated that one of the underlying mechanisms of *Panax japonicus* and *Panax notoginseng* formula might be associated with the promotion of angiogenic processes [28]. One study demonstrated that ginseng was capable of regulating antibody production by augmentation of *T* helper type 1 immune response (IL-2, IFN-gamma) and *T* helper type 2 immune response (IL-4, IL-10) cytokine production [29]. One study suggests that Radix Astragali extract (RAE) is a potent stimulator of angiogenesis and that it exerts its potential proangiogenic effects involving the VEGF and Akt-dependent signaling pathways [30]. Another review revealed that *Angelica sinensis* is an alternative treatment available for hair loss that acts through hair cycle pathways associated with hair follicle apoptosis regression during the catagen phase [31]. Adipose-derived mesenchymal stem cells (ADMSCs) can provide a promising future in the field of tissue engineering and regenerative medicine and may serve a wide variety of applications. A study showed that Rehmannia glutinosa oligosaccharide (RGO) might increase the viability and proliferative capacity and alleviate H_2_O_2_-induced apoptosis of human ADMSCs via the secretion of VEGF and hepatocyte growth factor. The results indicate that the application of RGO will enhance stem cell preservation and improve their therapeutic effects of cell therapy [32]. Various extracts and individual compounds derived from *Ligustrum lucidum* have been reported to possess a variety of pharmacological effects, e.g., immune regulation, antioxidative effect, antiageing effect, and anti-inflammation effect [33]. Another review also showed that *Ligustrum lucidum* could modulate estrogen receptor expression with no uterotrophic effect in ovariectomized rats [34] and improve bone quality in type 1 diabetic mice via stimulating parathyroid production [35]. Butanol extract from *Eclipta prostrata* has an antioxidative effect in rats, and saponin is a primary ingredient in butanol extract that has antioxidative effects in vitro [36].

From the above and previous information, we hypothesized that BT had a potential effect of improving hair growth via the induction of several growth factors (VEGF, EGF, and FGF), especially the expression of VEGF as the key mediator in hair growth development, physiological, and pathological processes. We also hypothesized that BT may have the potential effect for AGA through the immunomodulating effect that protects hair follicles from immune attacks and may have the potential effect for AGA through anti-inflammatory properties that protect hair follicles from inflammation. However, further studies are needed to evaluate this phenomenon.

Our previous randomized, double-blind placebo-controlled clinical trial revealed that in the BT treatment group, 52.9% of participants showed an increase in hair growth. The changes in hair growth are as follows: 5.9% slightly improved, 29.4% moderately improved, and 17.6% markedly improved [37]. This represents that the composition of formula mode is an important meaning for improving the research of treating diseases by integrated syndrome differentiation via the new clinical trial design [38].

In traditional Chinese medicine culture, qi (also chi) is an active principle forming part of any living organism. *Panax japonicus* could offer a sweet flavor and warming property effects, strongly invigorate primordial qi and is the principal herb formula [38]. Interestingly, a study indicated that Shengfaling Tincture acts to increase blood circulation and hair growth in rats with hair depilation. This study also expressed a significant reduction in blood viscosity and hematocrit in qi-deficiency rats who received Shengfaling treatment [39]. Danggui Buxue Tang (DBT) is an ancient Chinese medicinal decoction containing *Astragalus membranaceus* and *Angelica sinensis* (weight ratio of 5 : 1) that is being commonly prescribed as hematopoietic medicine to treat women with menopausal symptoms [40]. Pharmacological results indicate that DBT can stimulate the production of hematopoietic growth factor erythropoietin (EPO) [41]. Another study suggests that DBT improves immune function through the regulatory effects on immune function parameters, such as TNF-*α*, IL-12, NO production, and NF-*κ*B -mediated immune response [42]. We hypothesized that BT might have another potential effect to improve hair growth via increasing blood flow and qi.

Additional therapies for hair loss will become available in the future (e.g., topically effective Chinese herbal medicine). A better understanding of the causes and pathophysiology of alopecia should lead to more successful treatments involving the use of natural supplements that alter hair follicle cycling that provides protection from hair follicle immune attack. Given the accessibility of the follicle and the available liposome preparations for their ability to target the follicle, the topical gene therapy approach seems feasible [43]. A change in diet or taking the appropriate herbal health products or natural supplements will correct this widespread problem, need for further development in this area. Therefore, there will be more studies that will attempt to better understand the molecular mechanisms of hair-growth-promoting effects of BT.

## 5. Conclusion

We suggest that BT had possessed superior hair growth effectiveness through VEGF expression, and VEGF is considered as the most important mediator for the process of angiogenesis involved in hair growth development. This study presented a higher area percentage in the change of the number of hair follicles in the treatment group as compared to the control group. It was revealed that the BT has the potential effect of stimulating hair growth via upregulation of VEGF expression.

## Data Availability

The results are collected from basic research studies shown in the manuscript.
